# 219. Robust and Consistent Immune Response of the Adjuvanted Respiratory Syncytial Virus (RSV) Prefusion F Protein Vaccine (RSVPreF3 OA) Across Different Age Ranges and Frailty Status in Older Adults

**DOI:** 10.1093/ofid/ofae631.077

**Published:** 2025-01-29

**Authors:** Isabel Leroux-Roels, Robert G Feldman, Raffaele Antonelli-Incalzi, Dong-Gun Lee, Alberto Papi, Michael G Ison, Marie-Pierre David, Carline Vanden Abeele, Magali de Heusch, Nathalie De Schrevel, Catherine Gerard, Aurélie Olivier, Marie Van Der Wielen, Veronica Hulstrøm

**Affiliations:** Ghent University and Ghent University Hospital, Ghent, Belgium, Ghent, Oost-Vlaanderen, Belgium; Senior Clinical Trials Inc., Laguna Hills, CA, United States, Laguna Hills, California; Università Campus Bio-Medico di Roma, Rome, Italy, Rome, Lazio, Italy; The Catholic University of Korea, Seoul, South Korea, Seoul, Seoul-t'ukpyolsi, Republic of Korea; University of Ferrara, St. Anna University Hospital, Ferrara, Italy, Ferrara, Emilia-Romagna, Italy; National Institutes of Health, Derwood, MD; GSK, Wavre, Belgium, Wavre, Brabant Wallon, Belgium; GSK, Wavre, Belgium, Wavre, Brabant Wallon, Belgium; GSK, Wavre, Brabant Wallon, Belgium; GSK, Rixensart, Belgium, Rixensart, Brabant Wallon, Belgium; GSK, Rixensart, Belgium, Rixensart, Brabant Wallon, Belgium; GlaxoSmithKline Biologicals, Wavre, Brabant Wallon, Belgium; GSK, Wavre, Belgium, Wavre, Brabant Wallon, Belgium; GSK, Wavre, Belgium, Wavre, Brabant Wallon, Belgium

## Abstract

**Background:**

In a phase 3 study, the approved RSVPreF3 OA vaccine, administered as a single dose in adults aged ≥ 60 years, has demonstrated high efficacy against RSV disease and was well tolerated with a favorable safety profile. Here we present immunogenicity results in older adults of different age and frailty status from the same study.

**Figure:**
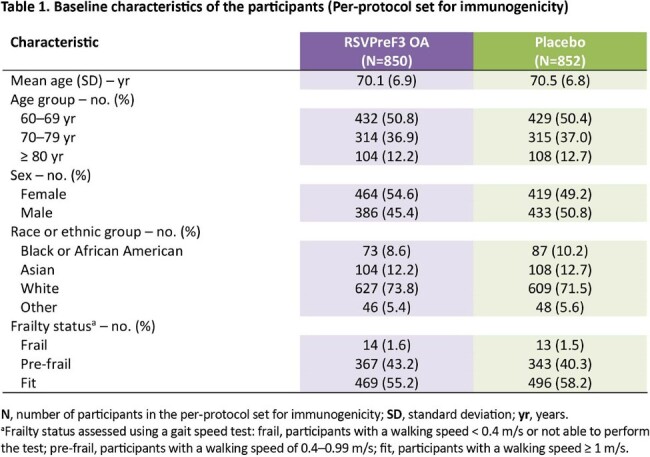

**Methods:**

This phase 3, placebo-controlled, observer-blind, multi-country study (NCT04886596) enrolled adults aged ≥ 60 years who were randomized (1:1) to receive a dose of RSVPreF3 OA or placebo, before the RSV season. Blood samples were collected at pre-vaccination (day 1) and 1 month post-vaccination (day 31). Humoral immune responses were assessed in a subset of participants, and outcomes included RSV-A and RSV-B neutralization titers by age category and frailty status.

**Figure:**
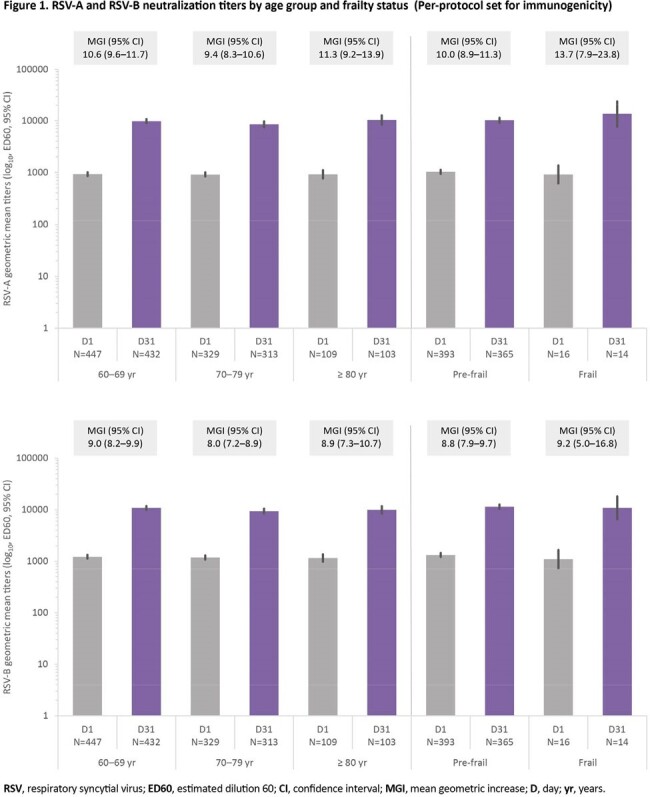

**Results:**

Of the 24,966 participants who received RSVPreF3 OA or placebo at day 1, 1,702 were included in the per-protocol set for immunogenicity. Demographic characteristics in the immunogenicity subset were well balanced between groups (**Table 1**). The mean age was 70.3 (± 6.8) years. At day 1, all tested participants had detectable RSV-A and RSV-B neutralization titers due to previous exposure to RSV. At day 31, 1 month post-vaccination, neutralization titers were between 9.4–11.3-fold (RSV-A) and between 8.0–9.0-fold (RSV-B) higher than pre-vaccination levels across the different age categories (**Figure 1**). In pre-frail and frail adults, neutralization titers increased 10.0- and 13.7-fold (RSV-A), and 8.8- and 9.2-fold (RSV-B) between day 1 and 1 month post-vaccination (**Figure 1**).

**Conclusion:**

For both RSV subtypes, RSVPreF3 OA induced a large increase in neutralization titers across the different age categories, including a robust immune response even in adults aged ≥80 years, known to have immunosenescence. A robust immune response was also observed in pre-frail and frail adults, however the results for frail adults should be considered with caution as the number of frail adults in the study was low.

**Funding:** GSK

**Disclosures:**

**Isabel Leroux-Roels, PhD MD**, Curevac: Grant/Research Support|GSK: Grant/Research Support|Icosavax: Grant/Research Support|Janseen Vaccines: Advisor/Consultant|Janseen Vaccines: Board Member|Janseen Vaccines: Grant/Research Support|Moderna: Grant/Research Support|MSD: Advisor/Consultant|MSD: Grant/Research Support|OSE Immunotherapeutics: Grant/Research Support|Osivax: Grant/Research Support **Robert G. Feldman, MD**, GSK: Payment to attend congress and speaking events, travel expenses for these events **Raffaele Antonelli-Incalzi, MD**, GSK: Grant/Research Support **Alberto Papi, MD**, Agenzia Italiana del farmaco (AIFA): Grant/Research Support|AstraZeneca: Advisor/Consultant|AstraZeneca: Board Member|AstraZeneca: Grant/Research Support|AstraZeneca: Honoraria|Avillion: Advisor/Consultant|Avillion: Board Member|Avillion: Honoraria|CHIESI: Advisor/Consultant|CHIESI: Board Member|CHIESI: Grant/Research Support|CHIESI: Honoraria|Edmond Pharma: Advisor/Consultant|Edmond Pharma: Honoraria|Elpen Pharmaceutica: Advisor/Consultant|Elpen Pharmaceutica: Board Member|Elpen Pharmaceutica: Honoraria|GSK: Advisor/Consultant|GSK: Board Member|GSK: Grant/Research Support|GSK: Honoraria|IQVIA: Board Member|IQVIA: Honoraria|Menarini: Honoraria|Mundipharma: Honoraria|Novartis: Advisor/Consultant|Novartis: Board Member|Novartis: Honoraria|Sanofi: Advisor/Consultant|Sanofi: Board Member|Sanofi: Grant/Research Support|Sanofi: Honoraria|Zambon: Advisor/Consultant|Zambon: Honoraria **Michael G. Ison, MD MS**, Adagio: Advisor/Consultant|Adamis: Advisor/Consultant|Adamis: Board Member|ADMA Biologics: Advisor/Consultant|AlloVir: Advisor/Consultant|AlloVir: Board Member|Cidara: Advisor/Consultant|CSL Behring: Board Member|Genentech: Advisor/Consultant|GSK: Grant/Research Support|ISIRV AVG: Chair|Janssen: Advisor/Consultant|Janssen: Board Member|Merck: Board Member|NIH: Board Member|Roche: Advisor/Consultant|Sequiris: Board Member|Shinogi: Advisor/Consultant|Takeda: Advisor/Consultant|Takeda: Board Member|Talaris: Advisor/Consultant|Talaris: Board Member|Transplant Infectious Disease: Editor-in-Chief|UpToDate: Royalties **Marie-Pierre David, Master in Statistics**, GSK: As GSK employee, I’m part of a patent application|GSK: Salary as GSK employee with stock options|GSK: Stocks/Bonds (Public Company) **Carline Vanden Abeele, MS**, GSK: Salary as GSK employee with stock options|GSK: Stocks/Bonds (Public Company) **Magali de Heusch, PhD**, GSK: Salary as GSK employee with stock options|GSK: Stocks/Bonds (Public Company) **Nathalie De Schrevel, PhD**, GSK: Magali De Heusch|GSK: Stocks/Bonds (Public Company) **Catherine Gerard, PhD**, GSK: Salary as GSK employee with stock options|GSK: Stocks/Bonds (Public Company) **Aurélie Olivier, PhD**, GSK: As GSK employee, I’m part of a patent application|GSK: employee|GSK: Stocks/Bonds (Public Company) **Marie Van Der Wielen, MD**, GSK: As GSK employee, I’m part of a patent application|GSK: Salary as GSK employee with stock options|GSK: Stocks/Bonds (Public Company) **Veronica Hulstrøm, MD, PhD**, GSK: Salary as GSK employee with stock options|GSK: Stocks/Bonds (Public Company)

